# Application of the rapid ethical assessment approach to enhance the ethical conduct of longitudinal population based female cancer research in an urban setting in Ethiopia

**DOI:** 10.1186/s12910-018-0328-8

**Published:** 2018-11-14

**Authors:** Alem Gebremariam, Alemayehu Worku Yalew, Selamawit Hirpa, Abigiya Wondimagegnehu, Mirgissa Kaba, Mathewos Assefa, Israel Mitiku, Eva Johanna Kantelhardt, Ahmedin Jemal, Adamu Addissie

**Affiliations:** 1 Department of Public Health, College of Medicine and Health Sciences, Adigrat University, Adigrat, Ethiopia; 2 School of Public Health, College of Health Sciences, Addis Ababa University, Addis Ababa, Ethiopia; 3 Department of Radiotherapy Center, School of Medicine, Addis Ababa University, Addis Ababa, Ethiopia; 4 Weema International, Addis Ababa, Ethiopia; 5 Institute of Medical Epidemiology, Biostatistics and Informatics, Martin-Luther-University, Halle, Germany; 6 Surveillance and Health Services Research, American Cancer Society, Atlanta, GA USA

**Keywords:** Rapid ethical assessment, Research ethics, Consent process, Cancer patients, Ethiopia

## Abstract

**Background:**

Rapid Ethical Assessment (REA) is an approach used to design context tailored consent process for voluntary participation of participants in research including human subjects. There is, however, limited evidence on the design of ethical assessment in studies targeting cancer patients in Ethiopia. REA was conducted to explore factors that influence the informed consent process among female cancer patients recruited for longitudinal research from Addis Ababa Population-based Cancer Registry.

**Methods:**

Qualitative study employing rapid ethnographic approach was conducted from May–July, 2017, at the Tikur Anbessa Specialized Referral Hospital. In-depth and key informants’ interviews were conducted among purposively selected 16 participants. Regular de-briefings among the study team helped to identify emerging themes and ensure saturation. Interviews and debriefings were tape recorded in Amharic, and transcribed and translated to English. Coding of the transcripts was facilitated by use of NVivo software. Thematic analysis was employed to respond to the initial questions and interpret findings.

**Results:**

Perceived barriers to voluntary study participation included lack of reporting back study results of previous studies, the decision making status of women, hopelessness or fatigue in the patients, shyness of the women, data collectors approach to the patient, and patient’s time constraints. Most of the patients preferred oral over written consent and face-to-face interview over telephone interview. Provision of detail information about the study, using short and understandable tool, competent, compassionate and respectful enumerators of the same gender were suggested to assure participation. Due to the perceived severity, the use of the term “cancer” was associated with fear and anxiety. Alternatively, uses of phrases like “breast or cervical illness/disease” were suggested during patient interviews.

**Conclusions:**

Voluntary participation is not straight forward but affected by different factors. Using competent, compassionate and respectful enumerators, short and precise questioning tools to limit the time of the interview could improve voluntary participation. Moreover, careful consideration of the patients and families concept of the disease such as wording and information has to be taken into account. This assessment helped in improving the consent process of the ongoing project on breast and cervical cancer patients.

## Background

Informed consent is one of the cornerstones of research and clinical ethics in healthcare [1, 2]. Obtaining informed consent from participants before enrolment in a study is an internationally accepted standard [3, 4]. Informed consent should address the voluntariness, capacity, disclosure, understanding and decision of the participants [5, 6]. Individual’s decision to participate should be made without coercion or undue influence. It is also important to consider capacity related to an individual’s ability to make decisions that stems from his or her ability to understand the information provided. Prospective research participants should get all relevant information about the research, including its nature, purpose, risks and potential benefits as well as the alternatives available [7].

In developing countries, study participants’ socioeconomic status, education level as well as health conditions have been found to render them vulnerable [8, 9], and is challenging on how to deliver complex information at the right time, in an appropriate language and style [10]. Studies conducted in various African countries showed that there are compromised levels of understandings among study participants about voluntary participation [11].

The consent process for cancer research becomes more complex when research is conducted among a group of people for whom it is difficult to discuss about cancer because of perceived severity of the disease and its lasting effect; often patients perceive the diagnosis of cancer as a death sentence [12, 13]. Approaching patients with cancer is not an easy task in clinical practice, because it may involve transmitting the medical information about their diagnosis, often unfavorable prognosis, risks and benefits of treatment, in addition to the possibility of disease progression and death [14]. Once diagnosed with cancer, stigma can negatively affect medical decision making, and medical care provision [15]. All the mentioned factors therefore can adversely affect the dynamics of the informed consent process among cancer patients taking part in research.

Given the adverse perceptions regarding cancer and high vulnerability of patients with cancer, it is of paramount importance that researchers understand the context and design setting of the consent process to improve voluntary participation in cancer related researches like breast and cervical cancer [11]. This can be done using Rapid Ethical Assessment (REA) which is testified as an important step for improving the research consent process in developing countries including Ethiopia [16–21]. Thus, this study was conducted to explore the determinants of and approach to gaining informed consent for cancer related researches in Ethiopia and other parts of Africa, and discuss the practical ways in which this information will be used to design the consent process for subsequent study entitled “Breast and cervical cancer patients’ experience in Addis Ababa Ethiopia: a follow-up study”, which is a longitudinal study aimed to document breast and cervical cancer patients’ experience from symptoms to diagnosis, treatment, and survivorship/mortality in Addis Ababa city, Ethiopia.

## Methods

### Study design and setting

This study was conducted among cancer patients attending Tikur Anbessa Specialized Referral Hospital (TASH), in Addis Ababa city. The study employed ethnographic research design with mix of methods to collect data. In-depth and key informants’ interviews were conducted with 16 purposively selected groups of respondents. The first group of participants was cancer patients (breast or cervical cancer) while the second group were relatives of breast or cervical cancer patients. These participants were residents in Addis Ababa city for more than 6 months, speak the local language and be conversant with the local culture, and have rich information about the area. The third group was health professionals who have been providing health services to cancer patients and have experience in cancer related studies. In addition, these groups were observed for their informed consent related interaction. The number of cancer patients, patients’ relative, and care providers included in the study were 6, 5, and 5, respectively. The number of study participants for each of the interview groups was determined based on theoretical saturation of data assessed through regular debriefings and interim analysis of data by the REA team.

### Data collection methods

Before data collection, arrangements were made with the REA team to learn more about the project, to explain the REA process, and to adapt interview tools. Literatures recommend including multidisciplinary researchers in the REA team [20, 22]. Therefore, the REA team for this study was composed of the Principal and two co-investigators of the “Breast and cervical cancer patients’ experience in Addis Ababa city: a follow-up study” project, a social scientist, and three public health experts. The team was responsible for developing and reviewing the interview guide, identifying In-depth Interview (IDI) participants, conducting the interview, making debriefing, organize data through recording and labeling, translation and transcription of collected data, doing interim analysis and provide feedback to the principal investigators of the project.

Interview guide was developed and discussed among the REA team. Different interview guides were used for the three target study groups. Data were collected by the REA team. All except one of the interviews were taped in addition to scribbled taken during the interview. Each of the interviews took 45 min on average. At the end of each day, the REA team debriefed on the main findings with an objective to identify emerging themes and issues for further exploration, to plans for the subsequent day’s data collection, and verifies saturation of information.

### Data quality assurance

Recall bias was mitigated by restricting the study participants to those patients who were diagnosed within one year range from the date of interview. Transferability of the findings was ensured by using person and method triangulations. Moreover, multidisciplinary REA team was formed, then oriented on REA, trained on the qualitative interview methods, repeated and intense meetings of REA members before and during the conduct of the REA to finalize tools and debrief was conducted. This improves the credibility of the collected data. Besides, the field notes, all interviews were tape recorded to grasp all the points during the interview. Moreover, debriefing among the data collectors and investigators was made on daily bases. Except one interview, all transcripts of the interviews were checked for errors by listening back to the audio-recording and reading the transcripts simultaneously. Computer based data coding was used to facilitate the reduction of the qualitative data without missing the central idea.

### Data analysis

Preliminary data analysis was in line with daily debriefings and reflections among the REA team members throughout the data collection and analysis period. However, detailed analysis with transcription, translation and detailed coding of the text data was done after completion of data collection.

All transcripts were entered into computer assisted qualitative data analysis software called NVIVO 11. Once the first cycle coding was completed, a second cycle coding (thematic analysis) was done based on the codes, and the findings were used for writing a scientific report for further documentation and dissemination. Quotes that best described the various categories and expressed what was said frequently in several groups were chosen.

### Ethical statement

Ethical clearance was obtained from the Institutional Review Board (IRB) of Addis Ababa University, College of Health Sciences. Interviewers obtained written informed consent of the respondents prior to the interviews. Participation in the study was completely voluntary and refusal to respond to some of the questions or interruption from the study was possible. All the information obtained from the respondents remained confidential and anonymous. Finally, the dissemination of the finding does not refer specific respondents but the general source population.

## Results

### Socio-demographic characteristics of participants

Six female cancer patients: 5 breast and 1 cervical cancer, and five relatives of cancer patients (3 breast and 2 cervical) were included in the in-depth interview (Table 1). The median age of the study participants was 37 years ranged from 20 to 77 years.

**Table 1:** Socio-demographic characteristics of IDI participants in Tikur Anbessa Specialized Referral Hospital, Oncology Clinic, Addis Ababa, Ethiopia, 2017

Participants code	Participant type	Educational	Marital status	Occupation	Diagnosis of the patient
P01	Patient	6th grade completed	Married	Housewife	Breast Ca
P02	Patient	8th grade completed	Single	No job	Breast Ca
P03	Patient	12th grade completed	Divorced	Guard	Breast Ca
P04	Patient	Read and write	Divorced	Small scale merchant	Breast Ca
P05	Patient	Read and write	Widowed	No job	Breast Ca
P06	Patient	Cannot read and write	Divorced	No job	Cervical Ca
R01	Relative	Diploma	Married	Agriculture extension	Breast Ca
R02	Relative	6th grade completed	Single	Daily laborer	Breast Ca
R03	Relative	3rd year University Student	Single	Student	Breast Ca
R04	Relative	TVT/ 10 + 3	Single	Student	Cervical Ca
R05	Relative	9th grade completed	Married	Day care for children	Cervical Ca

*Ca* Cancer, *TVT* Technical and Vocational Training, *P* Patient, *R* Relative

Moreover, in-depth interview with five key informants was made. The key informants include nurses and oncology residents who were working in the oncology clinic of TASH, and have experience on research or data collections (Table 2).

**Table 2:** Characteristics of the key-informants in Tikur Anbessa Specialized Hospital, Oncology clinic, Addis Ababa, Ethiopia, 2017

Participants code	Educational	Service year in the current department
HP01	BSc. Nurse	2 years
HP02	BSc. Nurse	2 years
HP03	MD + R4	more than a year
HP04	MD + R4	5 months
HP05	BSc. Nurse	4 years

*BSc* Bachelor of Science, *HP* Health Professionals, *MD* Medical Doctor, *R4* Resident year 4

### Participants’ awareness of research

Except one, all of the in-depth interviewees ever participated in research related interviews in their life. They tried to define research as it is something related to investigation done for the purpose of education, understanding the risk factors, causes, symptoms, nature of the diseases, reasons of delayed health seeking and improving its treatment.

One of the cancer patients, however, argued differently as research is something conducted for the benefit of the researcher whereas nothing to do for the interviewee and the community.

*“I just wanted to help you guys… I understand it is for your own educational purpose. … I did not give it any attention. It is for your own educational purpose (P01: BC patient)”.*

The key informants defined research as exploring or identifying better solution for a given problem in the community.

*“It is a broad term…It is a research which prioritizes problems and finds useful solutions by efficient resource utilization (HP03: Oncology Resident 4)*”.

“*To reduce the burden and improve the outcome…to investigate in detail about the causes and identify prevention mechanism (HP05: BSc., in nursing)”.*

### Participation in research

Most of the in-depth interviewees were aware of voluntary participation. Some of the respondents explained saying as *“Just like the way you did it now (P02: BC patient)****”*** which was referring to getting the willingness to participate in the study without any undue influence made by external body.

***“*** *We call it voluntary participation (It is) just the way you called me earlier. We call it voluntary if an individual participates without anyone’s pressure after knowing about the study (R04: patient relative)”.*

Similarly, the key informants explained voluntary participation as it is the involvement of the study participants based on independent informed decision made by the eligible study participants.

*“We cannot make participants involve on a study unless they are willing even though they fulfill the criteria (HP04: oncology resident 4).”*

### Barriers to voluntary participation

Some participants said that there should be no barrier to participate particularly in these types of cancer studies due to the perceived severity of the disease in the community and their willingness to contribute in alleviating such a “deadly disease”.

**“...** *. I do not think ... they have any barrier except the fear of the disease. No one will disagree to this. Even her husband will not disagree … (R02: patient relative)”.*

The following things, however, were reported as possible barriers to voluntary participation in cancer researches:

1. Dissatisfied to previous involvements or health service received: Participants who were unhappy with the feedback received in their past interview could consider as a waste of their time. Besides, dissatisfaction with the health service might hinder their voluntary participation
*“Some might not get the results after it is completed. Thus, there are different reasons. I have seen many people coming to the place I work at currently. Sometimes doctors come and take samples but we never received any feedback. It will be left there (R01: patient relative)”.*
*“… Usually research conducted here for masters, PhD but there is nothing done as a result of the research… (HP05: BSc. nurse)”*
2. Considering the interview as a tiresome: Some of the participants considered interview or research as a tiresome activity

*“Some people might say I am too old to go through such hustle (P05: BC patient)”.*

*“It might not be convenient and some people might not want to go through the trouble (R01: patient relative)”.*

3. Hopelessness or fatigue in the patients: Lack of hope in the patients due to the perceived severity of their illness (cancer), or the fatigue resulted from the treatment they took or the lasting symptom of their illness was noted as a barrier to voluntary participation.

*“… They all cannot be happy because the disease itself makes you unhappy ... They will be depressed psychologically by thinking that they have offensive body odor especially cervical cancer patients. They give up hope and become easily exhausted (HP02: BSc. nurse).”*

4. The decision making status of women: Some of the women could fail to decide to participate in a study by themselves due to the women’s’ level of decision making power, and may need approval from their partners before deciding to participate in a study.
*“If you have a bad husband he might be one of the barriers. Your family takes the lead in this issue. There are families which let you be more outgoing (P01: BC patient).”*
*“In my surrounding women have lower decision-making capacity? Many women spend their time doing household work at home. Male/husband decides most of the things… (R02: patient relative)”.*
5. Shyness of the women: women are shyer than men making them less self-expressive.

*“Most of them work at home and they are too shy to go outside (R02: patient relative)”.*

*“They (women) might be shy and have lack of self-esteem which might be related to cultural issues. They have been under cultural influences since they were children thus they might fear to be interviewed (HP03: Oncology resident).”*

6. Household responsibility: women are busy with home responsibilities. As a result, they might not want to spend time by involving themselves in the interview. Moreover, some may also fail to give attention to the interview, thinking the activities they have in their home.

*“They might not have the time. They have a lot of things to take care of including their home, children and personal life. This is one of the barriers (P01: BC patient).”*

7. Approach to the patient: Failure to keep the norms of the community could dissatisfy the respondent and could be a barrier to participation.

*“… The interviewer might say some culturally and socially insensitive things. It is not good if you cannot communicate well with the respondent. For example, if you interview an elderly by saying ‘Anta’ rather than ‘Antu’* 1 *he/she might feel disrespected. You should make sure that you are using the right name prefixes while interviewing Sheki, pop etc… (HPO3: oncology resident).”*

#### Suggested actions to improve voluntary participations

For the question, *what should be done to improve voluntary participation,* many of the cancer patients and relatives responded *‘as you did it now’*, and forwarded the following possible solutions or actions which should be done to increase voluntary participation of potential study participants.

1. Information about the study: The participants recommended as the consent sheet should include information on the burden and severity of the disease, the importance and the objectives of the study, success stories of previous studies, anticipated harms from the study, confidentiality of the information obtained, the procedure of the study, the right to withdraw from the study, and provision of compensation. The participants emphasized not only inclusion, but the data collectors should make sure the participants understood and comprehend the information before asking their willingness.

*“You should not ask for a signature at first. I am here because I am willing. Hence you should tell others that the study is useful for them and for you. Moreover, you should tell them that it will help you to see the disease in a broad perspective and to identify another treatment (P01: BC patient).”*

*“You should tell them about the study’s objective explicitly and asking them for their consent. We should try to make them willing by understanding the objectives well not because they are ashamed to say no (R03: patient relative).”*

*“We need to show them the impact of the research which has been done before … (HP05: BSc. nurse)”*

2. Competent enumerators: Participants recommended that enumerators should be expert in the area of study so that they can address questions raised by the study participants during the interview. Moreover, the data collectors should master the questionnaire before going to the interviewee, be familiarized with the data collection setting including the venue for interview, and create rapport with the respondent.

*“… I went somewhere to collect data and the questions were about how many eggs the respondent eats and the like. Then the respondent insulted me and told me that she could eat if she can have one (Laugh)… (R01: patient relative).”*

*“We should also consider the venue of the interview. For example, there is no one around; you are doing this interview in private. Thus, the place of the interview should be separate so that the patient answers all your questions without being uncomfortable... (HP01: BSC. nurse).”*

*“First it is better to come before the period of data collection and check their appointments and diagnosis which will help you to counsel the patient before the actual data collection (HP02: BSc. nurse).”*

3. Compassionate and respectful approach: Some of the study participants also noted as the data collectors should be respectful and able to politely invite the eligible study participants, understand the patients feeling, stabilizing them first, give the participants chance to ask the data collector.

*“First, you should ask her about her disease and if there is anything, you can help her with. They might not be willing if you just ask the questions (P01: BC patient).”*

*“The researcher should try to comfort the patient and make him/her understand about the disease. Moreover, he/she should support patients and be there for them so that they do not feel sad because of the disease. You should tell the patients to relax and come to you for support. (R05: patient relative)”.*

*“The researcher should be very friendly, greet the patient, show support, ask how he/she is doing etc. If you simply try to ask the patient about the study, they will refuse to give you the information. We can try to help them and ask them about their diagnosis and how he/she is feeling… (HP02: BSc. nurse).”*

4. Tool comprehension: They also recommended the importance of making the research tool simple, understandable and short to avoid prolonged interview.

*“Make the interviews short so that they do not lose interest (P02: BC patient)”.*

*“I suggest making the questions as simple and short because they might get exhausted and do not respond well. It is better to make the questionnaire short without losing its objective (HP01: BSc. Nurse)”.*

5. Compensation: Three of the key informants raised the need of provision of compensations and or pre-informing if you are going to provide money as compensation to the time or transportation the participants could spend.

“*We need to give them incentive (money) for their transportation cost and so on….They are extremely poor. We need to support them (HP05: BSc. nurse)”*

*“…We have to tell them if they benefit anything including payment and transportation (HP04: Oncology resident)”.*

### Consent types and participants preference

Most of the participants preferred oral consent over written consent. They justified this as written consent needs reading and comprehending ability of the participant which is difficult for most of the study participants. Moreover, it demands participants confidence, education and detail explanation on the study by the interviewer because signature is related with something serious and considered as it increase the future accountability and responsibility of those who signed. Some of the participants also noted as requesting written consent could induce the participants to provide biased (only positive) responses.

While we approached the study participants for this study, some of the study participants did not want to give their signature a head of our interview though they agreed to participate in the interview. They do not feel comfortable when asked to sign on the paper even after agreed to the interview.

*“I am volunteer to be interviewed. Why I should give my signature while I already agreed to interview (P01: BC patient)?”*

*“I think the signature might be a bit difficult. It is not the same when you talk to him/her orally and when you ask for a signature. I am not saying this because you asked me for a signature; I do not mind doing that. We all have different perspectives … Because they think that it will cause them some problems (P03: BC patient)”.*

If signature is must, eight of the participants suggested and preferred the signature to be collected at the end of the interview. They reasoned out as this could create minimal concern in the participants because at the end of the interview the participants would have already become clear with the content of the interview and can give their signature freely. For instance, when some of the cancer patients asked to put their signature after we explained them about the study, they responded as:

*“It is ok I will sign it later… first let see how this goes… I want to know how it goes before I sign the consent (P01: BC patient)”.*

*“I wanted to have more information before I sign … I wanted to know what the questions are first (P02: BC patient).”*

*“If there should be a signature, it is better to make it at the end because they might not be open about their responses if you ask for a signature at first. The best approach is to carry out the interview without signature. … The data collector should take oral consent first without telling the participant that he/she have to sign but at the end, the interviewer might get a signature for the reason that the participant already gave the required information… (HP01: BSc. nurse).”*

On the contrary, four of the respondents argued for the importance of getting written signed consent over oral consent which increases the retention of the study participants over the follow-up period, and as a more reliable evidence of voluntary participation. They said signature should be taken before the commencement of the interview, after adequately explaining and creating trust with the respondent at the very beginning.

*“…Patients are usually willing (to participate) but they do not understand the real importance of the study... We can just explain the general idea of the research and they will not be unhappy when we ask them for signature and name… Signing is not a new thing for cancer patients. They sign for chemotherapy, radiotherapy and to give consent for operation procedures. Thus, they will not be shocked just because you ask them for signature (HP04: Oncology resident)”*

*“I signed for you after you explained it to me. Would I sign for you if I did not understand what it is? It does not matter if it is at the beginning or the end, people will sign if they understand. No one will sign without knowing what he/she is signing for… You can ask for the signature after you explain about the study but before going through the questionnaire. For example, I signed the consent before the interview right? (R02: patient relative).”*

Two respondents mentioned that the consent type should be individualized because the participants’ choice could depend on the behavior of the individual. As a solution they recommended making the consent process flexible and providing both options to the participant to choose. Lastly, one of the cancer patient believed that the patients should sign without hesitation because the health professionals do not harm the patients. She explained as:

*“You are the professional so it is their duty to sign or they have to sign… They have to sign and participate on the study. That is my opinion (P04: BC patient).”*

#### Preference on gender of the interviewer

Enumerator’s gender was not reported as a prominent problem for the intended project but almost all of the study participants noted the importance of using same sex interviewer especially for addressing sensitive issues like sexuality related questions. They exemplify this by using female interviewers for the cervical cancer surveys.

*“It would not matter to me if the interviewer is male or female. But women might be more open to other women hence it would affect their openness … In my opinion it is better to use female interviewers. So, that they share their story openly and wouldn’t fear (R04: patient relative).”*

*“I am not sure. For example, regarding the current study, it doesn’t matter if it is male or female because there are no questions related with sexual history. I do not think it would matter if the interviewer is either one of the two sexes but it might be better if we use female interviewer to ask cervical cancer patients (HP01: BSc. nurse).”*

Two of the key informants rather emphasized the importance of using health professionals working in the facility who have experience working with cancer patients where the study participants are recruited. They justified this as the data collectors could help address the questions or concerns raised by the patients during the interview.

*“The patients always have an actual complain thus it is better to be interviewed with an individual who can assist them or have communication with someone who can help them. A data collector from outside might not give attention or may unintentionally neglect their complaints. However, professionals from the facility are familiar with these complaints thus; they might pass on patients to someone who can help them even so they cannot help them directly… (HP04: oncology resident).”*

#### Attitude towards repeated follow-up interviews

Most participants confirmed as there is no problem with repeated interval interview of breast and cervical cancer patients, but emphasized on the importance of the decision making power of the women and their busy schedule, and compensation for their travel expenses if they come for the purpose of the study, and the timing of the interview to be conducted versus their home and outdoor responsibility of the women. Three of the participants, however, responded as repeated interviews could be difficult because of participants fatigue and exhaustion. Two of the respondents argued against feasibility of making phone interview in the consecutive interviews.

*“They might say yes if they do not have anything to do at that time… They will come to the place you want them to if they do not have anything to do during the interview period… They might come if they are not busy and finish their work (R03: patient relative).”*

*“No, they will not be willing to get interviewed repeatedly. They don’t even be willing, when we ask them again to fill out what has been missed in our interview (HP05: BSc. nurse)”*.

*“No, you cannot use the phone. They will not accept that unless you use face-to-face interview. Forget the phone… How would you know what it is …For example, I would not give you any information by phone…, they might not remember you and feel confused. If it was I, I might not remember you and even ask you who you are (R01: patient relative)”.*

### On participants’ recruitment

When the participants selected from the waiting room of patients, during the selection of the eligible study participants the unselected women could have a sort of disturbance, feel wonder, unhappy, and think as the study participants are purposely selected for treatment. The participants explained this as below:

*“There might be mothers who are unhappy or fussy about it … I might wonder why they want to interview that person (P02: BC patient)”*

### Participants’ expectations after interviewed for the research

Many of the participants said nothing as an immediate return to the interview. Besides, the long term effect of the study, some participants could expect money, favor in getting treatment, advice and answer to their concern immediately after completion of the interview. One also stated as they could have fear of exposing their responses and diagnosis to Mass-Medias or others. One of the key informant interview also revealed as the women could took it as part of their treatment due to the setting where the interview is taking place.

*“I expect you to be successful and see the patients cured soon. At least the future generation should be saved from this (P03: BC patient).”*

*“She might think that the interview could be released on media. Moreover, she might fear that they may call her name... She might fear that other people would know about her disease if she hasn’t told anyone else but it is fine if she already told people (R03: patient relative).”*

*“Phone number, to support them throughout the process, to ask many questions, to shorten them their appointment date, and expect them to support them in all aspect (HP05: BSc. nurse)”*

### Use of the word ‘cancer’

Most of the study participants including the health care professionals interviewed noted the difficulty of using the term cancer with patients and their relatives. This is due to the perceived severity of cancer in the community equating it to death and social stigma linked to cancer in the community. The health professionals felt difficult of declaring the patients’ diagnosis. As a result, it was mentioned that there were cancer patients who do not know their diagnosis while taking cancer treatment. Thus, using the word cancer during the interview could be a bad news to the patient and induce psychological disturbance in the study participants. As a solution, instead of using the phrase “your breast or cervical cancer”, these participants suggested using phrases like: “your breast or cervical illness”, “breast or cervical case”, and “abnormal body growth of your breast”. Some of the participants also recommended questioning the patients to explain why they visited the health facility, and to name the diagnosis of their illness before commencement of the interview. Moreover, one of the interviewee stressed the importance of knowing the circumstance of the cancer treatment center before approaching the patient for interview. They mentioned about the importance of communicating with family members to clarify the levels of awareness of patients about their diagnosis.

*“Try to make the participants talk about it by themselves. Ask her what diseases she has instead of telling her she have cancer. (P01: BC patient)”.*

*“The patients usually do not feel comfortable if you use the term cancer repeatedly. From my experience, they become uncomfortable when you use the term cancer, so it is better not to use it. First, you just ask the patient why they come here. Some of them tell you that they are here for radiotherapy because that is what they had been told. If you ask them about their diagnosis, they might tell you that they have a breast or cervical illness (HP01: BSc. nurse).”*

*“The disease (Yeh besheta), your pain (Hememo) (HP05: BSc. Nurse)”*

*“That is what I meant earlier when I say that the researcher should know the cancer center well. There might be patients who do not know they have cancer. For example, if an attendant asks us not to tell his/her mother that she has cancer and if the data collector interviews the patient without the presence of the attendant using the word cancer repeatedly, he/she might disclose the case unknowingly. Thus, it is better to understand the situation around here. The data collector might not know whether each patient he/she comes across with knows about his or her diagnosis but it is better to be cautious while using the word cancer. It is not that it is appropriate not to tell the patient the diagnosis based on the current health care service, it is the status of most patients, which makes it difficult. Thus, it needs being mindfulness (HP04: oncology resident).”*

Nevertheless, some of the participants also reflected as there is no problem of using the term cancer during interviewing the patients for the research. Rather, they pointed out the importance of discussing about cancer openly explaining as the patients are already living with it and as this could help to create awareness about cancer in the patients and community.

*“I think it is better to be open about it because the disease is spreading. It is everywhere. At least one person got the disease from the entire family members. Thus, I would like it if we talk about it openly and educate the community (P03: BC patient)”.*

*“The participants already know the disease why would they worry. For example, I know about my diseases thus it is my duty to answer your questions in detail (P05: BC patient).”*

## Discussion

Conducting rapid ethical assessment prior to implementing the actual data collection was found useful to design understandable and setting specific consent process in earlier studies conducted in Ethiopia and other countries [17–21]. This study was the first urban based REA. It helped to identify important issues which need to be carefully addressed in the course of approaching and recruiting study participants for the proposed study.

Acknowledging voluntary participation, as participating in a study without undue influence; the participants have raised different barriers and also solutions for increasing the voluntary participation of potential study participants. Participants noted as eligible study participants could underestimate the importance of a study and hinder their voluntary participation. As a solution, most of the study participants suggested the need of detail information on the study. Thus, before asking the willingness of the study participants, the data collectors should able to provide comprehensive information about the study, and also able to make sure the participants comprehension. The quality of informed consent process depends on the adequacy of the information provided [23], and the participants comprehension of the information [5, 6, 24, 25]. Otherwise, failure to know the right to withdraw from the study for instance, could increase participants’ denial to our invitation or induce involuntary participation [26].

Enumerators approach could also determine voluntary participation of study participants. The enumerators should able to communicate to the respondents using non-technical and understandable language. For instance, comprehending the term ‘research’ by the study participants is crucial to understand the goal of the study and increase their voluntary participation. Unless, the ambiguity of understanding about research is addressed, it could affect their willingness to participate and the validity of the information planned to be received from the study participants.

The term “cancer” is considered fatal and incurable disease by both the cancer patients and relatives interviewed. This view was also documented in a study conducted in Northern Ethiopia [27]. As a result of this, it was not simple to use the word cancer while interviewing some of the breast and cervical cancer patients. Such difficulty was also reported by the health professionals interviewed. More surprisingly, some of the participants did not know as they have cancer even while they were taking cancer treatment because their family members inform to the providers not to tell to the clients as their illness is cancer. Besides, some of the patients do know like the word cancer though they know their diagnosis is cancer. As a solution, the participants suggested using other phrases like “your breast/cervical illness or disease”, and letting the patients to talk about the reason for their visit to the health facility and using that phrase. This indicates lack of open discussion about cancer in the community due to the perceived severity of cancer. In such circumstance, provision of appropriate consent and declaring the patients’ diagnosis for the purpose of the research could result in conflict with the interest of the patients’ family, physician and the researchers [28]. Some of the participants noted as this could aggravate the problem of open discussion among the patients and the community. They suggested talking openly to increase awareness about cancer in the patients and the community. This indicate a communication gap, and health professionals who are helping cancer patients need to work on mechanisms of disclosing such sensitive diagnosis to the patients and relatives.

Individuals sign an informed consent document to authorize their agreement to participate in a study. Most of the in-depth and key informants, however, preferred oral consent while even they agreed to participate in the study. This finding was concurring with the studies conducted in different parts of Ethiopia [17, 27, 29, 30]. The participants relate signature with legal accountability and could hinder participants from responding freely for any question [27, 30]. Some of the participants recommend collecting written consent at the end of the interview. This could be in part the reflection of inadequacy of the information given and low comprehension [17], and the literacy level of the study participants [31]. This was also witnessed by some of the respondents who believed in written consent as far as the participants received, and understood the right information about the study. We can understand from this, the enumerators should able to explain in detail and ensure as the participants’ comprehended what he/she explained. Nigussie et al., 2016, documented as written consent demands more explanation and understanding of the study by the study participants [30].

Prior to entering to the actual interview questions, the interviewee should build thrust on the interviewer to give his/her written consent and a genuine response. But, this was not in many of the cases. If written consent is must, many of the study participants suggested or preferred the signature to be collected at the end of the interview; after participants knew what the interview is all about.

The role of enumerators’ gender, participants’ selection, level of decision making to participate and expectation after interviewed should be considered and adequately addressed during the design of the consent process of the study. The respondents noted the importance of using preferable female enumerators working in the health facility, be cautious during picking the eligible study participants from the pull of patients waiting for treatment and addressing the possible participants’ expectations.

Efforts were made to maintain the credibility and dependability of the study findings using multidisciplinary research team and involving different groups of interviewees however, the transferability of this finding might not achieved due to the small sample size used and the socio-cultural difference of the study subjects in this metropolitan city. We have tried to interview recently diagnosed patients; however, recall of past events with a foresight of experience may unconsciously make the stories of these women biased.

## Conclusions

This REA pointed out potential barriers and solutions which may be considered to improve voluntary participation. Using competent, compassionate and respectful enumerators, short and precise questioning tools to limit the time of the interview could improve voluntary participation. Moreover, careful consideration of the patients and families concept of the disease such as wording and information has to be taken into account. This assessment helped us in improving the consent process of the ongoing project on breast and cancer patients.

## Abbreviations

AAPBCR: Addis ababa population based cancer registry
IDIs: In-depth interview
PI: Principal investigator
REA: Rapid ethical assessment
TASH: Tikur anbessa specialized hospital

## Footnotes

1 Anta/Antu (Amharic language - Ethiopian National/working language): socially acceptable form of salutation addressing someone older or higher authority to show respect and regard.
